# Individualized Target Fortification of Breast Milk with Protein, Carbohydrates, and Fat for Preterm Infants: Effect on Neurodevelopment

**DOI:** 10.3390/nu17111764

**Published:** 2025-05-23

**Authors:** Niels Rochow, Nicolas Gabriel Leier, Gisela Adrienne Weiss, Gerhard Fusch, Anaam Ali, Akshdeep Bhatia, Salhab el Helou, Jan Däbritz, Christoph Fusch

**Affiliations:** 1 Department of Pediatrics, Paracelsus Medical University, Breslauer Str. 201, 90471 Nürnberg, Germany; niels.rochow@klinikum-nuernberg.de (N.R.); nicolas@leier.net (N.G.L.); gisela.weiss@klinikum-nuernberg.de (G.A.W.); 2 Department of Pediatrics, McMaster University, Hamilton Health Sciences, Hamilton, ON L8S 4L8, Canada; gefusch@mcmaster.ca (G.F.); anaamali@gmail.com (A.A.); akshdeep.bhatia@mail.utoronto.ca (A.B.); elhelos@mcmaster.ca (S.e.H.); 3 Department of Pediatrics, Rostock University Medical Center, 18057 Rostock, Germany; 4 Department of Health Research Methods, Evidence and Impact, McMaster University, Hamilton, ON L8S 4L8, Canada; 5 Department of Orthopedic Surgery, University of Toronto, Toronto, ON M5T 1P5, Canada; 6 Department of Pediatrics, Greifswald University Medical Center, 17475 Greifswald, Germany; jan.daebritz@med.uni-greifswald.de; 7 Department of Pediatrics, Klinikum Westbrandenburg Potsdam, 14467 Potsdam, Germany; 8 Institute of Clinical Research and Systems Medicine, Health and Medical University, 14471 Potsdam, Germany

**Keywords:** preterm infants, target fortification, human milk, milk analysis, fat, protein, carbohydrates, neurodevelopment, growth, body composition

## Abstract

**Background/Objectives**: Preterm infants are at high risk of extrauterine growth restriction and suboptimal neurological development due to cumulative nutrient deficits. Standard fortification (SF) of human milk does not account for individual macronutrient variability, potentially leading to inadequate intake. Target fortification (TFO) adjusts supplementation based on the measured macronutrient content, aimed at providing macronutrient intake aligned with ESPGHAN (European Society for Paediatric Gastroenterology, Hepatology and Nutrition) recommendations and optimize growth and development. This study aims to evaluate the effects of TFO compared to SF on growth, body composition, and neurological outcomes at 18 months corrected age. **Methods**: In this double-blind, randomized controlled trial, preterm infants (<30 weeks gestation) received either SF or TFO for at least three weeks. Macronutrient levels in breast milk were analyzed three times per week, with modular adjustments in the TFO group. Growth parameters, body composition at 36 weeks postmenstrual age, and Bayley Scales of Infant and Toddler Development III (BSID-III) scores at 18 months corrected age were assessed (n = 69). **Results**: TFO significantly increased protein, fat, and carbohydrate intake compared to SF, leading to higher weight gain (2514 ± 289 g vs. 2283 ± 332 g, *p* < 0.01) and growth velocity (21.7 ± 2.3 g/kg/d vs. 19.2 ± 2.2 g/kg/d, *p* < 0.001). In infants whose mother’s milk had low protein levels, fat-free mass was significantly higher with TFO compared to SF. BSID-III scores were higher in the TFO group across cognitive, language, and motor domains, with significant improvements in expressive language scores in infants whose mother’s milk had high protein levels (*p* < 0.05). The number of preterm infants with a motor BSID-III score of ≤70 was significantly lower in the TFO group compared to the SF group (0 vs. 3, *p* < 0.05). **Conclusions**: TFO enhanced growth and body composition and may support better neurological outcomes in preterm infants. While most BSID-III differences were not statistically significant, the data suggest that TFO may reduce the risk of developmental delays. Larger, multicenter trials are needed to confirm these findings.

## 1. Introduction

Preterm birth represents a critical challenge in neonatology, as these infants experience a highly vulnerable period of development outside the womb. Ensuring adequate postnatal growth and neurological development is essential, as suboptimal growth trajectories have been linked to adverse long-term outcomes, including increased risks of metabolic disorders and impaired cognitive function [1,2]. Despite advances in neonatal care, extrauterine growth restriction remains common, particularly in very-low-birth-weight infants, due to accumulated nutritional deficits [3]. While traditional fortification strategies aim to improve nutrient intake, they often fail to meet the individual requirements of preterm infants, potentially affecting both short- and long-term outcomes.

The human brain undergoes a significant growth spurt from mid-gestation to two years postnatally, with critical microstructural processes such as oligodendrocyte differentiation and axonal growth occurring in the third trimester of pregnancy [4]. Preterm infants miss this critical in utero phase, placing them at risk of suboptimal brain development. Adequate macronutrient intake, particularly protein, is crucial in supporting this rapid neurological maturation. Evidence suggests that a higher intake of macronutrients and fat-free mass gain is associated with improved neurological outcomes, whereas excessive fat mass gain may not have the same benefits [5,6].

Breast milk is the preferred source of nutrition for preterm infants due to its immunological and developmental benefits, including bioactive growth factors, phospholipids, and probiotics [7,8]. However, its macronutrient composition is highly variable, fluctuating based on maternal factors, lactation stage, and time of day [9]. Standard fortification (SF), the most widely used fortification strategy, involves adding fixed doses of commercial fortifiers to breast milk. However, SF does not account for individual macronutrient variations. This leads to inconsistent nutrient intake and persistent growth deficits, with up to 58% of preterm infants experiencing postnatal growth restriction despite fortification [10]. Other individualized fortification methods, such as adjustable fortification, attempt to mitigate the deficits of SF by modifying protein intake based on an infant’s metabolic markers, such as blood urea nitrogen. However, this still lacks direct measurement of actual macronutrient intake, leading to reactive rather than proactive adjustments [11]. As a result, both standard and adjustable fortification may allow nutritional deficits to accumulate before they are recognized and corrected, potentially compromising early growth and development.

Target fortification (TFO) is an individualized fortification method that offers a more precise approach by measuring macronutrient levels in breast milk and adjusting protein, fat, and carbohydrate supplementation accordingly. This method aligns nutrient intake with the recommendations of the ESPGHAN [12] and reduces nutrient variability across feedings. Unlike SF or adjustable fortification, TFO ensures that each infant receives optimized nutrition rather than milk with an unpredictable composition. Our previous research has demonstrated that TFO leads to improved weight gain, growth velocity, and metabolic parameters while reducing feeding intolerance, with these outcomes measured during and at the end of the intervention period [13]. However, it remains unclear whether these short-term improvements in growth and body composition translate into meaningful long-term benefits, particularly in neurological development, which is among the most critical outcomes for preterm infants. 

The biological rationale for TFO’s potential benefits on neurodevelopment lies in its ability to provide a more stable and balanced nutrient supply compared to SF. Unlike SF, TFO minimizes day-to-day fluctuations in macronutrient intake and more reliably meets recommended macronutrient targets. Evidence suggests that even with constant caloric intake, greater variability in nutrient supply can adversely affect metabolic responses [14], and preterm infants, with their immature metabolism, may be particularly vulnerable. By replicating the steady nutrient environment of in utero development, TFO may prevent intermittent nutrient shortfalls during critical periods of brain growth. Furthermore, TFO optimizes the protein-to-energy ratio, promoting lean mass and brain tissue growth rather than protein oxidation for energy, as shown by Kashyap et al. [15]. Imbalanced nutrition during neonatal intensive care unit (NICU) care can later be compensated for fat mass but not as effectively for gains in fat-free mass, length, or organ mass, including brain mass, potentially leading to lasting deficits in neurodevelopment.

While existing studies have focused primarily on immediate post-intervention effects, the potential for TFO to influence neurodevelopmental trajectories has not yet been explored. By reducing macronutrient variability and preventing cumulative nutrient deficits, we hypothesize that TFO will enhance both short-term growth and long-term neurological outcomes. This study aims to evaluate the effects of TFO compared to SF on growth, body composition, and neurological outcomes at 18 months corrected age. Specifically, it investigates whether TFO results in higher Bayley Scales of Infant and Toddler Development III (BSID-III) scores, improved weight and length gain, and better body composition at 36 weeks postmenstrual age (PMA). The findings of this study could provide crucial insights into optimizing neonatal nutrition and improving the developmental trajectory of preterm infants.

## 2. Materials and Methods

### 2.1. Study Design

This prospective, single-center, double-blind, randomized controlled trial (RCT) was conducted in a Level III NICU (McMaster Children’s Hospital, Hamilton, ON, Canada) from January 2012 to December 2016. This nutritional study includes the analysis of both short-term and long-term treatment outcomes. The analysis of short-term outcomes has already been published [13]. The present study examines the neurological development of preterm infants at 18 months corrected age as a long-term follow-up outcome. For the study population assessed using the BSID-III, the short-term outcomes were reanalyzed. Preterm infants were fed either standard-fortified or target-fortified human milk. The control group was defined as the group receiving only SF, while the intervention group received SF plus TFO. The local ethics committee approved the study (Hamilton Integrated Research Ethics Board, McMaster University, Hamilton, Canada; Reference No. 12-109, Approval Date: 9 July 2012). The study was registered on 30 May 2012, at ClinicalTrials.gov (identifier: NCT01609894). Informed written consent was obtained from both parents. The study was conducted in accordance with the CONSORT guidelines for randomized controlled trials.

### 2.2. Inclusion and Exclusion Criteria

Infants eligible for this study were those born at a gestational age of <30 weeks, as determined by maternal records or early fetal ultrasound. To be included, they had to have an enteral intake of ≥100 mL/kg/d for ≥24 h. Additionally, the intervention was required to last for ≥3 consecutive weeks after achieving full enteral feeding, defined as a daily intake of ≥150 mL/kg. In the case of multiple births, each infant meeting the inclusion criteria was included in the study, with siblings randomized individually to one of the study arms.

Infants were excluded if they presented with intrauterine growth restriction or were classified as small for gestational age, defined by a birth weight below the 3rd percentile according to sex-specific reference data [16]. Those with gastrointestinal malformations, severe congenital anomalies, or chromosomal abnormalities were not eligible. Conditions such as enterostomy, short bowel syndrome, or prolonged fluid restrictions (<140 mL/kg/d for ≥3 consecutive days) led to exclusion. Infants receiving ≥25% of their caloric intake from formula over a one-week period were also excluded. Medical conditions such as Gram-negative sepsis, necrotizing enterocolitis (NEC, Bell stage 2 or 3), kidney disease with symptoms such as oliguria, anuria, proteinuria, or hematuria, alongside elevated creatinine levels (≥130 mmol/L) [17] or blood urea nitrogen levels (≥10 mmol/L) [18], and liver dysfunction defined by jaundice (direct bilirubin level >1.0 mg/dL) with abnormal liver enzymes (aspartate aminotransferase, alanine aminotransferase, or gamma-glutamyl transferase) met exclusion criteria. Participation in another clinical trial that could influence study outcomes or a high likelihood of transfer to another NICU or Level II pediatric unit outside McMaster Children’s Hospital also resulted in exclusion.

### 2.3. Randomization and Blinding

Randomization was stratified by gestational age (<28 weeks; ≥28 weeks) in blocks of varying sizes (2, 4, and 6) to minimize allocation bias. A series of opaque, sealed, consecutively numbered envelopes were prepared for each stratum and opened by dietitians in their offices outside the NICU. Apart from the dietitians, all study personnel were blinded to the randomization and nutritional intervention. The fortification prescription was determined by attending physicians in both study arms. The added modular components did not alter the appearance of the milk [13].

### 2.4. Measurement of Nutrient Concentration in Native Human Milk

Native human milk was pooled in 24 h batches, and 2.5 mL samples were collected. Before analysis, the samples were homogenized for 15 s using an ultrasonic disintegrator (VCX 130; Chemical Instruments AB, Sollentuna, Sweden). Osmolality was measured in both native and fortified human milk using an osmometer (3320 MicroOsmometer; Advanced Instruments, Norwood, MA, USA). Macronutrient analysis was conducted three times per week using a calibrated and validated near-infrared bedside milk analyzer (SpectraStar; Unity Scientific, Brookfield, CT, USA) [19,20].

To calculate actual daily intake for data analysis, nutrient content was measured before fortification in all batches from both study arms. Protein and fat content were analyzed using the validated near-infrared bedside milk analyzer, while lactose content was determined using an established ultra-performance liquid chromatography–tandem mass spectrometry reference method [19]. Enteral feeding volume was recorded daily to calculate total fluid and macronutrient intake. A chemical analysis was performed on 10% of samples to validate the milk analyzer results.

### 2.5. Nutritional Standards of Study Groups

TFO was calculated and administered by trained dietitians for each macronutrient using a standardized study recipe sheet [13]. Human milk analysis was conducted three times weekly, with previous values used on non-testing days [13].

The fortifier used was a commercially available standard fortifier (Enfamil HMF^®^, Mead Johnson, Cincinnati, Hamilton County, OH, USA; per 100 g powder: 38.7 g protein, 14.1 g carbohydrates, 35.2 g fat). Four packets of 0.71 g each were added to 100 mL human milk and provided an additional 1 g fat, 1.1 g protein, and 0.4 g carbohydrates. For TFO, the standard fortifier was used along with modular components: a polyunsaturated fat emulsion (0.5 g fat/mL; Microlipid, Nestlé HealthCare Nutrition, St. Louis Park, MN, USA), whey protein powder (0.86 g protein/g; Beneprotein, Nestlé HealthCare Nutrition, St. Louis Park, MN, USA), and glucose polymer powder (0.94 g carbohydrates/g; Polycal, Nutricia, Trowbridge, UK). Infants receiving donor milk were supplemented with an additional 0.4 g whey protein powder per 100 mL (Beneprotein), following McMaster NICU guidelines.

**Figure 1:**
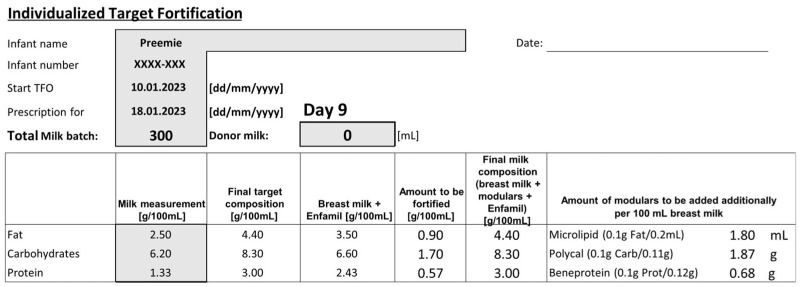
Calculation of TFO. Template used by dietetic staff to determine the required amounts of modular protein, carbohydrate, and fat supplements to achieve ESPGHAN-recommended targets when added to standard-fortified breast milk. TFO—target fortification.

In the SF and TFO groups, the standard fortifier was introduced gradually over two days once a total fluid intake of 120 mL/kg/d was achieved. Once the full SF concentration was reached in the TFO group, the modular components were gradually added over a period of three days. To prepare the target-fortified milk, SF was first added to native human milk in the recommended dosage. The modular components were then incorporated to meet the ESPGHAN-recommended intake levels of 4.4 g fat, 8.3 g carbohydrates, and 3.0 g protein per 100 mL, based on an average fluid intake of 150 mL/kg/d [12]. The amount of required modulars was calculated by subtracting the standard-fortified breast milk content for fat, protein, and carbs from the ESPGHAN-recommended targets. A model calculation is shown in Figure 1. Growth velocity was calculated from study day 2 after the complete introduction of TFO.

### 2.6. Assessment of Neurological Development

Neurological development was assessed at 18 months corrected age using the BSID-III. Cognitive, language (receptive and expressive), and motor (fine and gross motor) scales were analyzed based on US validation standards. Social–emotional and adaptive behavior scales were not included, as they rely on parental reports, which may introduce subjective biases. Furthermore, the primary focus of this study was on directly assessed neurocognitive and motor outcomes, which are considered more objective and closely linked to early brain development and nutritional influences. The mean composite BSID-III score is 100 (standard deviation = 15). Children with BSID-III scores ≤85, ≤70, and ≤55 were categorized as having mild, moderate, or severe developmental delay, respectively. Assessments were conducted by trained child psychiatrists at McMaster Children’s Hospital, with each test lasting approximately 90 min [21].

### 2.7. Evaluation of Outcome Parameters

Weight gain velocity (g/kg/d) was measured during the first 21 days of intervention. Weight was recorded every other day to an accuracy of 10 g using an electronic scale. Additionally, macronutrient intake, nutritional efficiency, head circumference, crown–heel length, major morbidities, and metabolic parameters (blood glucose, blood urea nitrogen, triglycerides) were assessed on days 7, 14, and 21. Neurological development at 18 months was correlated with these parameters.

Infants were stratified further into low-protein and high-protein groups within the control and intervention arms based on the projected protein intake after SF. This categorization aimed to better identify children at the highest risk of poor neurological development, particularly those in the low-protein group. The low-protein subgroup was calculated by analyzing the protein content of their native unfortified milk, and calculating the expected contribution from SF. Infants whose estimated protein intakes were below the median of the study population (3.5 g/kg/d) were classified as low-protein, and those with estimated protein intakes above as high-protein.

Nutritional efficiency (g/100 mL) was calculated as the ratio of daily weight gain (g/kg/d) to total fluid intake (mL/kg/d). Anthropometric measurements, including crown–heel length and front-occipital circumference, were recorded at admission and then weekly during the intervention period with an accuracy of 0.5 cm. Body composition, including fat mass and fat-free mass, was assessed at 36 weeks PMA before discharge or transfer using whole-body air displacement plethysmography (PEAPOD Infant Body Composition System, COSMED USA Inc., Concord, CA, USA). Daily quality control checks were performed according to the manufacturer’s instructions. All anthropometric and body composition measurements were conducted by two trained examiners [13]. The study was performed at a tertiary center, leading to a 30% retro-transfer rate to local level 2 hospitals before 36 weeks PMA. As a result, secondary outcome measurements such as body composition and growth at 36 weeks were not available for transferred infants. Among the 69 infants included in this neurological follow-up study, data on body composition and growth at 36 weeks PMA were missing for 18 infants, specifically, 6 in the control group and 12 in the intervention group.

### 2.8. Quality Control

Good Clinical and Laboratory Practice (GCLP) standards were applied to all analytical point-of-care instruments (blood gas analyzer, osmometer, milk analyzer) [22]. Daily quality controls were conducted for the near-infrared milk analyzer, and sample storage followed established protocols [19].

### 2.9. Statistical Analysis

The sample size calculations for this study were based on the expected short-term outcomes for the parameter of weight gain. To detect a difference in weight gain of 1.8 ± 3.1 g/kg/d, with a power of 80%, and a significance level of 5%, a sample size of 48 infants per group was determined to be necessary to reject the null hypothesis that the average differences in the intervention and control groups are equal [13]. The anticipated neurological treatment outcomes were not taken into account when determining the required sample size [13]. Statistical analyses followed CONSORT guidelines [23]. Descriptive statistics were reported as mean (standard deviation) or median (min–max) for continuous variables and count (%) for categorical variables. For the per-protocol analysis, only preterm infants who had received nutrition as specified in the study protocol for at least 14 days were included. Birth and study characteristics were compared between groups (control vs. intervention) using t-tests and chi^2^-test and the occurrence of pregnancy risk factors and severe morbidities in the NICU were compared between groups using chi^2^-test. The outcomes related to growth, body composition, nutritional parameters, and neurological development were compared as well between groups (control vs. intervention and low-protein vs. high-protein groups) using t-tests. The means of nominally scaled control variables, such as the number of children with BSID-III scores ≤85 or ≤70, as well as maternal and perinatal risk factors, were analyzed for systematic differences between the control and intervention groups using the chi^2^-test. Logistic regression analyzed the relationship between baseline characteristics and outcomes, while linear mixed models assessed longitudinal associations between intervention groups, nutrition, and body composition. All statistical tests were two-tailed with a significance level of 0.05, using SPSS (v26; IBM, New York, NY, USA) and R (v4.1.3; R Foundation, Vienna, Austria).

## 3. Results

### 3.1. Recruitment Process of Participants

**Figure 2:**
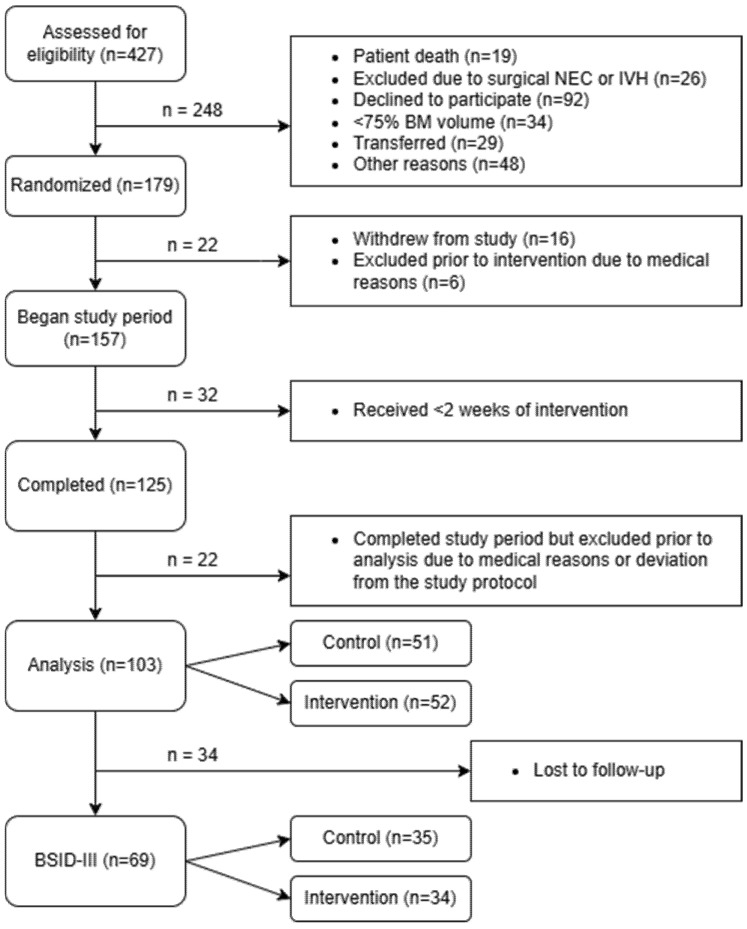
CONSORT flow diagram of the recruitment process. BM—breast milk; BSID-III—Bayley Scales of Infant and Toddler Development III; IVH—intraventricular hemorrhage; NEC—necrotizing enterocolitis.

Of a total of 427 infants that were assessed for eligibility (January 2013 to September 2016), 103 preterm infants (control group: n = 51, intervention group: n = 52) completed the intervention according to the protocol and were included in the analysis [13]. Among these 103 preterm infants, 69 underwent follow-up assessments using the BSID-III at 18 months corrected age (control group: n = 35, intervention group: n = 34) (Figure 2).

### 3.2. Patient Characteristics

The baseline characteristics of the participating preterm infants (Table 1) showed no significant differences between the control and intervention groups, regardless of whether the analysis included all infants who completed the intervention (n = 103), only those assessed with the BSID-III (n = 69), or only those not assessed with the BSID-III (n = 34). For these three populations, comparisons were made based on birth characteristics, including anthropometric data, sex, Apgar scores, gestational age at birth, and maternal age, as well as study-related characteristics such as PMA, days of life at study initiation, and duration of the intervention [24]. Additionally, for infants assessed with the BSID-III, anthropometric data and age at the time of the BSID-III evaluation were compared.

Regarding pregnancy risk factors and severe morbidities in the NICU, no significant differences were found between groups, with two exceptions. Pregnancy-induced hypertension or preeclampsia was more common among mothers in the intervention group whose infants underwent BSID-III assessment. Additionally, feeding disorders were less prevalent in the intervention group (Table 2).

### 3.3. Nutritional Characteristics

In the infants assessed with the BSID-III, there were no differences between the intervention and control group regarding the fat, protein, and carbohydrate content of the analyzed native human milk. However, the intervention group had a significantly higher average intake of fat, carbohydrates, and protein, as well as a significantly higher protein-to-energy ratio (Table 3).

Fat intake in both the control and intervention groups, at 7.1 ± 0.7 g/kg/d and 7.6 ± 0.7 g/kg/d, respectively, exceeded the ESPGHAN recommendation of 4.4–6.8 g/kg/d. Carbohydrate intake in the control group was slightly below the ESPGHAN recommendation (11.6–13.2 g/kg/d) at 10.8 ± 0.8 g/kg/d, while the intervention group slightly exceeded the recommendation at 13.6 ± 0.6 g/kg/d. Protein intake in the control and intervention groups, at 3.6 ± 0.3 g/kg/d and 4.5 ± 0.2 g/kg/d, respectively, was at the lower and upper limits of the ESPGHAN recommendation (3.5–4.5 g/kg/d).

In the subgroup analysis, the low-protein group in the control arm did not reach the recommended protein intake, with an average of 3.3 ± 0.2 g/kg/d, whereas the high-protein group in the control arm achieved the recommendation at 3.9 ± 0.2 g/kg/d. In the intervention group, protein intake for both the low- and high-protein subgroups was at the upper limit of the recommendation, with 4.5 ± 0.1 g/kg/d and 4.6 ± 0.2 g/kg/d, respectively.

**Figure 3:**
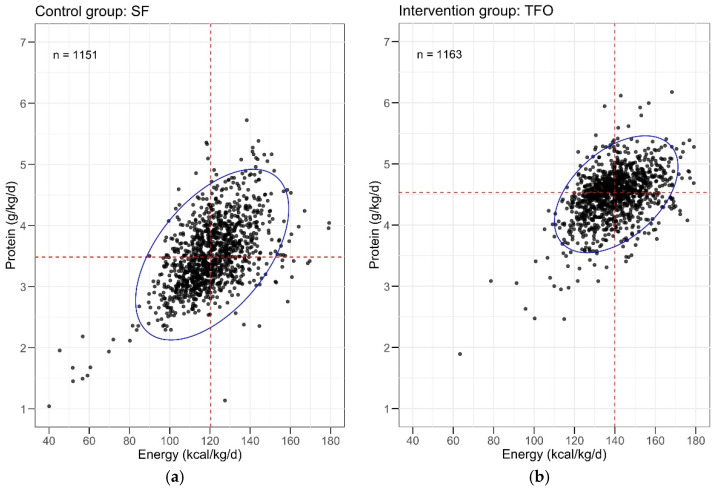
Protein and energy intakes after fortification of human milk. Protein intake in g/kg/d and energy intake in kcal/kg/d had been calculated for every infant daily. Values were plotted against each other and are shown for (**a**) the control group (n = 1151) and (**b**) the intervention group (n = 1163). Blue ellipse marks the 95% confidence interval. A smaller 95% confidence interval represents smaller variability in protein and/or energy intake. Red dashed lines – median, n—number of measurements; SF—standard fortification; TFO—target fortification.

The 95% confidence intervals (CI) for protein and energy intake per kilogram and day were significantly smaller in the TFO group compared to the SF group (Figure 3). TFO notably reduced macronutrient variability and minimized the number of extreme outliers with highly unbalanced nutritional composition.

### 3.4. Short-Term Effects of the Intervention

At 36 weeks PMA, body weight in the intervention group was significantly higher than in the control group (2514 ± 289 g vs. 2283 ± 332 g, mean difference [95% CI] of 231 g [81, 381], *p* < 0.01). Growth velocity during the first 21 days of the intervention (21.7 ± 2.3 g/kg/d vs. 19.2 ± 2.2 g/kg/d, mean difference [95% CI] of 2.5 g/kg/d [1.4, 3.6], *p* < 0.001) and nutritional efficiency (14.3 ± 1.7 g/dL vs. 12.4 ± 1.6 g/dL, mean difference [95% CI] of 1.9 g/dL [1.1, 2.2], *p* < 0.001) were also significantly higher in the intervention group.

In the subgroup with low native human milk protein content, TFO led to significantly improved growth (body weight 2525 ± 283 g vs. 2134 ± 277 g, mean difference [95% CI] of 391 g [203, 578], *p* < 0.001), while the weight difference in the high-protein subgroup was not significant.

Body composition measurements at 36 weeks PMA showed no significant differences in fat mass or fat-free mass between the control and intervention groups in the high-protein subgroup. However, in the TFO subgroup with low protein content, fat mass (610 ± 191 g vs. 451 ± 144 g, mean difference [95% CI] of 159 g [23, 295], *p* < 0.05) and fat-free mass (1920 ± 183 g vs. 1691 ± 240 g, mean difference [95% CI] of 229 g [54, 404], *p* < 0.05) were significantly higher (Table 4).

### 3.5. Evaluation of BSID-III Scores

Neurological development assessed using BSID-III at 18 months showed higher mean cognitive, language, and motor scores in the intervention group compared to the control group. However, none of these differences were statistically significant for the main BSID-III scales (Table 5).

The highest cognitive BSID-III scores were observed in infants from the TFO subgroup with high protein content (102.5 ± 10.0), while the lowest cognitive scores (93.5 ± 15.9) were found in infants from the control group whose mothers had below-average human milk protein content. This difference was not statistically significant (Table 5).

Comparing preterm infants in the low-protein and high-protein groups, regardless of study group, showed a weak trend toward better scores in the high-protein group, though statistical significance was not reached (Table 5).

**Figure 4:**
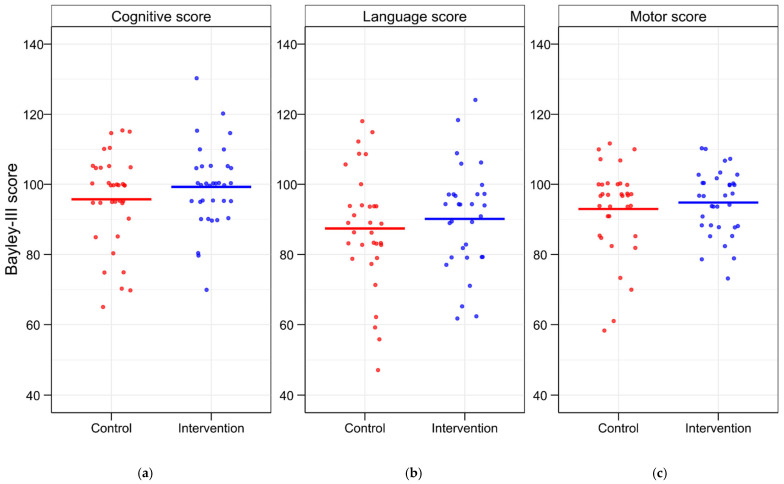
BSID-III scores for individual preterm infants. BSID-III scores were assessed on (**a**) the cognitive scale, (**b**) the language scale, and (**c**) the motor scale in the control and intervention groups. Values are presented as median and each point representing the BSID-III score of a single preterm infant. BSID-III—Bayley Scales of Infant and Toddler Development III.

No significant differences were found within the low-protein subgroup. However, in the high-protein subgroup, the intervention group showed significantly higher scores in both the expressive and overall language scales than the control group (Table 5). The number of infants with scores ≤85 and ≤70 was lower in the TFO group across all categories (Figure 4). A score of ≤55 was observed in only one infant from the control group on the language scale. Notably, the number of preterm infants with a motor BSID-III score of ≤70 was significantly lower in the intervention group compared to the control group. Three infants in the control group had motor scores ≤70 on the BSID-III, while no such cases occurred in the intervention group. This difference was statistically significant with *p* = 0.035 (Table 6).

### 3.6. Correlation Analyses of Growth and Neurological Development

Correlation analyses revealed that higher protein and lactose intake were significantly associated with increased growth velocity (*p* < 0.001). Fat intake had no impact on growth velocity.

Infants with a higher gestational age showed significantly better results across all three BSID-III scores, with cognitive scores reaching significance at *p* < 0.05 and language and motor scores at *p* < 0.01. Belonging to the intervention group or the high-protein group had a significant positive effect on cognitive scores (*p* < 0.05), while language and motor scores showed a positive trend, though not statistically significant.

Overall, a higher average total protein intake was associated with improved cognitive scores (*p* < 0.05). However, the reduced variability in protein intake during TFO compared to SF, as indicated by the standard deviation, had no significant effect on BSID-III scores. Gender as a confounding factor did not influence BSID-III scores.

### 3.7. Differences Between Short- and Long-Term Outcomes

**Figure 5:**
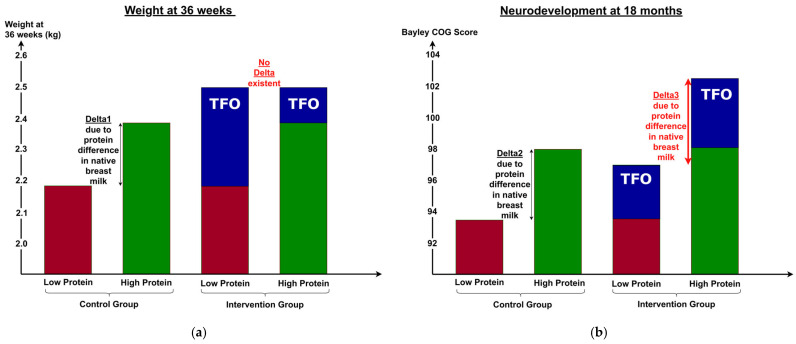
Comparison of the effect size of short- vs. long-term outcome. Effect size of (**a**) the short-term outcome weight at 36 weeks is compared to (**b**) the long-term outcome Bayley cognitive (COG) score at 18 months, displayed for the low-protein (red) and high-protein (green) subgroups within the control and intervention groups. The difference (blue) between intervention and control group is attributed to TFO. Delta1 and Delta2 can be explained by differences in the protein content of native human milk during the intervention; Delta3 is attributable to variations in the protein content of native human milk before the start of TFO and after discharge from the NICU. COG—cognitive, TFO—target fortification.

In the short term, TFO directly improved body weight, composition, and metabolic parameters by optimizing nutrient intake, particularly protein. Infants receiving SF had higher weights in the high-protein subgroup compared to the low-protein subgroup (see Delta1, Figure 5a). In contrast, infants receiving TFO achieved similar weight gain regardless of native human milk protein content, indicating that TFO effectively corrected nutrient intake differences and met ESPGHAN recommendations. Thus, infants born to mothers with low human milk protein content benefited significantly from TFO in terms of growth.

For long-term neurological outcomes, infants whose mothers had low-protein human milk showed little improvement with TFO, while those receiving high-protein human milk exhibited greater developmental gains (Figure 5b).

## 4. Discussion

This follow-up study examines neurological development at 18 months corrected age in preterm infants (<30 weeks gestation) who participated in a double-blind RCT by Rochow et al. [13]. In the original trial, infants received either target-fortified or standard-fortified human milk. The target-fortified milk was individually enriched, based on measurements of milk composition, with fat, protein, and carbohydrates using modular products. TFO provides macronutrient intake aligned with the ESPGHAN recommendations and resulted in improved growth, body composition, metabolic parameters, and reduced neonatal morbidity at discharge [13].

This study is the first to assess long-term neurological outcomes, showing a trend toward improvement across all BSID-III scales at 18 months.

### 4.1. Comparison with Previously Published TFO Studies

Few studies have investigated TFO in preterm infants, particularly its impact on neurological development. To date, in addition to Rochow et al. [13], 16 studies on TFO have been published [25,26,27,28,29,30,31,32,33,34,35,36,37,38,39,40]. Five of these studies lacked an RCT design with a defined control group. Among them, one study fortified only protein, two studies fortified protein and fat, and two studies fortified all three macronutrients [25,28,29,34,39].

Seven RCTs supplemented only protein in addition to SF [27,30,31,35,36,37,38]. These studies reported mixed results, ranging from no improvements in growth parameters to significantly improved growth. Growth velocities between 17.7 and 25.7 g/kg/d were achieved, compared to 21.7 g/kg/d in this study. Most studies showed an increased protein intake and protein-to-energy ratio with TFO, but without a proportional increase in energy intake, suggesting additional protein was metabolized rather than used for growth.

One RCT, which target-fortified only protein in the intervention group, assessed neurological development at 24 months corrected age using BSID-III (n = 22) [31]. This is the only TFO study that has published neurological development data. The intervention group had significantly higher cognitive and motor scores compared to the control group. The intervention group received modular protein fortification with a target of 4.5 g/kg/d. The control group received SF. No significant differences in growth velocity were observed, but preterm infants born before 27 weeks had significantly higher weight and length z-scores and increased fat-free mass at 32 and 35 weeks PMA.

Another study investigating the neurological development of preterm infants after TFO was discontinued due to increased feeding intolerance in the intervention group [40]. The authors suggested that the fortifier used lacked fat and increased osmolarity due to excessive modular fat supplementation. However, methodological concerns raise doubts about the study’s validity, including a high dropout rate, frequent issues with milk analyzers, and inconsistent baseline characteristics [40]. In contrast, the present study found fewer cases of feeding intolerance with TFO (5 vs. 11 in the control group).

An RCT comparing low (3.8 g/kg/d) vs. high (4.3 g/kg/d) protein intake found no growth differences despite higher protein intake in the high-protein group [32]. The blood urea nitrogen level in the high-protein group increased by 50%, indicating that excess protein was metabolized rather than used for growth. This reinforces the idea that energy intake was the limiting factor [41]. Another RCT fortified all three macronutrients but only supplemented protein modularly and fat and carbohydrates in fixed combinations to increase energy intake. No significant differences were observed in macronutrient intake between the SF and TFO groups, leading to no significant growth differences [33].

A study combining adjustable fortification with TFO found similar growth and nutrient intake between groups, possibly due to high blood urea nitrogen targets in the control group [26,42]. Additionally, only 25% of feedings consisted of human milk, and no infant was exclusively human milk-fed. Neurological outcomes at three years are still under investigation.

### 4.2. Interpretation of Results in the Scientific Context

TFO enabled macronutrient intake to align with ESPGHAN recommendations [12]. With SF alone, 50% of infants would not have met the lower target of 3.5 g protein per kg per day. TFO precisely achieved the upper target for protein intake of 4.5 g/kg/d, while the mean protein intake in the SF group was 3.6 g/kg/d. TFO reduced macronutrient variability and minimized the number of extreme outliers in human milk composition, an effect that only adding extra protein to fortified human milk would not achieve.

Higher nutrient intake is linked to improved growth and a lower rate of extrauterine growth restriction [43,44,45,46,47,48]. In this study, the intervention group had significantly greater protein, fat, carbohydrate, and energy intake, resulting in higher body weight, faster growth velocity, and improved nutritional efficiency [13]. In the low-protein subgroup, both fat mass and fat-free mass were significantly higher with TFO.

TFO also led to better neurological outcomes, with BSID-III scores at 18 months showing higher cognitive, language, and motor scores in the intervention group, though not statistically significant. Subgroup analysis revealed a significant improvement in expressive language scores in the high-protein group (90.4 ± 6.2 vs. 83.4 ± 10.3, *p* < 0.05). It should be noted, however, that this subgroup consisted of a relatively small number of infants (15 in the control group and 10 in the intervention group), and the observed differences may be due to chance. Therefore, this finding should be interpreted with caution and confirmed in larger, adequately powered studies. Compared to SF, TFO resulted in fewer preterm infants with BSID-III scores ≤85 and ≤70 across all scales, with a statistically significant difference in motor scores ≤70. Additionally, the only infant with a score of ≤55 on the language scale was found in the control group. Cognitive and motor development in early childhood are shaped by a wide range of interacting influences, such as genetic predisposition, family environment, and access to developmental support services. Therefore, the specific contribution of a nutritional intervention like TFO may be subtle and challenging to isolate within a relatively limited cohort. However, previous studies confirm that early protein and energy intake positively correlate with improved BSID scores and brain volume [49,50]. Early fat intake is also associated with improved neurological outcomes [51]. TFO allows us to identify infants at risk of neurological developmental delays while simultaneously addressing this risk. The goal of TFO is not to achieve “superfortification” but rather to identify human milk samples with low macronutrient content and ensure that every feeding aligns with ESPGHAN recommendations by adding modular components.

Growth velocity appears to have a significant, possibly independent impact on neurological development. This study’s observed effect size aligns with previous research, with the intervention group achieving an average growth velocity of 21.7 g/kg/d. Studies on extremely low-birth-weight infants have linked faster in-hospital weight gain with lower incidences of BSID-II scores of mental and psychomotor development indices <70 [52]. The best neurological outcomes were linked to average growth rates of 21.2 g/kg/d from regaining birth weight until reaching 2000 g. Faster weight gain from term to four months corrected age was also associated with better psychomotor development index scores [5].

Recent research attributes improved neurological outcomes to increased fat-free mass [6,53], which is tied to optimized calorie and macronutrient intake [54]. In this study, TFO significantly increased fat-free mass in the low-protein subgroup. Head circumference was also larger in this group (31.4 ± 1.4 cm vs. 30.6 ± 1.3 cm), a factor strongly correlated with brain size and cognitive outcomes [5,55,56].

While most BSID-III scale differences between the SF and TFO groups were not statistically significant, the study shows a clear trend favoring TFO. Given ongoing debates about the interpretation of *p*-values in biomedical research [57], the lack of statistical significance likely reflects the small sample size and large standard deviations rather than an absence of effect. Importantly, the sample size calculation for this trial was based on short-term weight gain outcomes, not long-term neurodevelopmental outcomes. A post hoc power analysis indicates that to detect a mean difference of 5 points in BSID-III scores (with an assumed standard deviation of 15, a power of 80%, and an alpha level of 0.05), a minimum of 142 infants per group would be required. Therefore, the current study has been underpowered to detect statistically significant differences in BSID-III outcomes, despite potentially meaningful effects. Moreover, even in the absence of statistical significance, a difference of 5 points on the BSID-III may be of clinical relevance, particularly when considering long-term developmental trajectories. It is well recognized that medical decisions are not made solely on the basis of *p* < 0.05 but also consider the magnitude, direction, and consistency of observed effects in the context of biological plausibility and clinical relevance. Thus, the trends observed in our study support the need for further investigation in larger, adequately powered trials to fully evaluate the impact of TFO on neurodevelopmental outcomes.

### 4.3. Differences Between Short- and Long-Term Outcomes

Short-term outcomes, such as body weight and composition at NICU discharge, differed from long-term neurological outcomes at 18 months corrected age. While short-term outcomes reflect in-hospital nutrition, long-term outcomes depend on both NICU and post-discharge nutrition.

TFO most benefited the low-protein group in terms of growth, as more protein was added to their human milk. However, this effect did not extend to neurological outcomes, suggesting that exposure to low-protein human milk before TFO initiation and after NICU discharge may have diminished its benefits.

**Figure 6:**
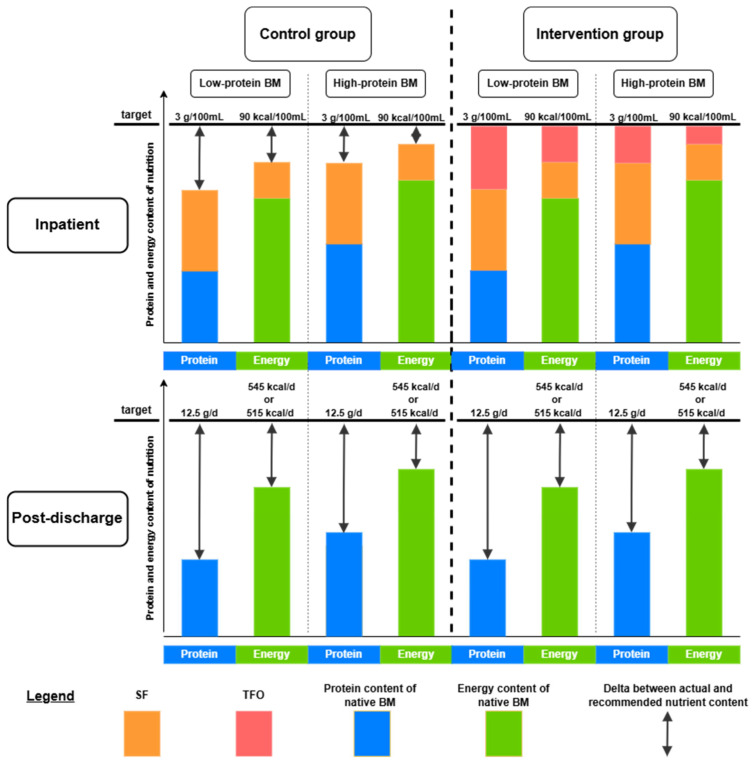
Measured protein and energy content of nutrition before fortification, after SF, and after TFO, as well as the expected protein and energy content of breast milk after discharge. The target values during the NICU stay align with the ESPGHAN guidelines, set at 3.0 g of protein per 100 mL and 90 kcal of energy per 100 mL, assuming a fluid intake of 150 mL/kg/d [12]. The post-discharge target values follow World Health Organization recommendations for newborns aged 0 to 3 months, with 12.5 g/d of protein and 545 kcal/d for male newborns and 515 kcal/d for female newborns [58]. BM—breast milk; SF—standard fortification; TFO—target fortification.

During hospitalization, the protein and energy deficits in human milk are partially corrected by SF and completely balanced by TFO. However, after discharge, human milk can no longer be adequately fortified, leading to the reaccumulation of protein and energy deficits (Figure 6).

The protein content of human milk naturally decreases over time [9], but it remains unclear whether mothers with initially low-protein human milk continued to produce it after discharge. If so, TFO could help identify these mothers early, enabling individualized post-discharge fortification plans.

Previous studies adding fixed fortifiers to human milk post-discharge showed no clear benefits for weight gain or neurological development, likely because they did not account for variations in human milk macronutrient content [59].

### 4.4. Strengths and Limitations of the Study

This is the first completed double-blind RCT evaluating neurological outcomes following individualized TFO of all three macronutrients. The findings have significant clinical implications, as TFO provides a feasible strategy to improve neurological development in preterm infants. The intervention resulted in better neurological outcomes, and TFO has proven practical both in this study and in previous research. Conducting three milk measurements per week represents a manageable cost–benefit ratio, adding 35–40 min of workload per infant weekly, which could be offset by improved outcomes and shorter hospital stays [20].

Baseline characteristics, nutritional characteristics, and short-term treatment outcomes were reanalyzed for the subgroup of preterm infants who underwent BSID-III testing. The results were consistent with the short-term findings previously published from the initial RCT [13]. Relevant confounding factors, such as maternal conditions and preterm morbidity, were assessed.

Human milk analyzers are medical devices and adhering to GCLP and performing proper device validation were essential for accurate macronutrient measurement. A multicenter quality initiative has shown that measurement errors can have clinical consequences [60]. Safety was ensured through daily quality controls, which must also be maintained in other institutions implementing TFO.

The sample size of the initial RCT was larger than most previous TFO studies, despite the study’s complex design and higher workload. However, as discussed above, the study was still underpowered to detect statistically significant differences in neurological outcomes, specifically BSID-III scores at 18 months.

A limitation of this study was the relatively high dropout rate at follow-up, with only 67% of infants from the initial study participating in BSID-III evaluation. Similarly, approximately 30% of the body composition data at 36 weeks PMA were missing due to retro-transfers to local Level II hospitals, which limited the availability of this secondary outcome and may impact the interpretation and generalizability of the body composition results. This high retro-transfer rate is a typical feature of neonatal care systems that are highly centralized, where very-preterm infants are initially cared for in a few specialized tertiary centers. Once infants are stable enough, they are often transferred to regional Level II hospitals closer to their families for ongoing care. In addition, when bed availability becomes limited, even locally born infants may be transferred to free up resources for new admissions.

Recruiting larger samples at a single center is challenging due to eligibility constraints, staff workload, and the long-term commitment required from parents. The single-center design may also restrict generalizability to other NICU settings. Clinical practices, patient populations, and resource availability can vary considerably across institutions and regions. This potentially affects the feasibility and outcomes of implementing TFO in different environments. To address these limitations, larger multicenter studies are an important next step.

Despite TFO, macronutrient variability was not completely eliminated because milk measurements and fortification adjustments were conducted three times per week instead of daily. For some macronutrients, particularly fat, the ESPGHAN upper limits were already exceeded after adding SF, resulting in occasional overfortification. Additionally, approximately half of the infants had unexpectedly high protein levels in their native human milk, affecting the results and necessitating subgroup analysis.

TFO was applied for an average of 29 days, starting on day 24 of life, meaning early nutrient deficits [3] could not be fully corrected, though further deficiencies were prevented.

Since this was the first RCT investigating TFO in preterm infants, ensuring safety and good tolerability was a priority. The study confirmed that TFO is safe and feasible in clinical practice. Extending the duration of TFO and implementing it earlier after birth could potentially enhance its benefits, which should be explored in future studies.

Post-discharge nutrition remains unknown, as no data were collected, and fortifiers were not routinely provided to parents. Given that rapid brain growth continues until the second year of life, future studies should track post-discharge intake, especially in infants receiving low-protein human milk.

BSID-III scores at 18 months provide only a moderate prediction of actual long-term cognitive abilities [61]. However, they remain the most widely used method for assessing preterm neurological development. Testing at 18 months allows for comparability between studies and was selected because it aligns with the routine follow-up schedule at McMaster Children’s Hospital. Adhering to this standard follow-up practice ensured a high rate of participation and consistency in assessments. While 18-month assessments can detect early developmental delays, it is acknowledged that some effects of early nutritional interventions may only become fully apparent at later stages, such as school age, when higher cognitive functions are more developed. However, assessing outcomes at older ages introduces a greater number of potential confounding factors, including differences in home environment, parenting styles, and access to educational and therapeutic interventions, which can obscure the direct impact of early nutrition. Future research should therefore continue to explore TFO’s impact on neurological outcomes at school age while carefully considering these additional variables.

While confounding factors such as gestational age, birth weight, sex, and neonatal diseases were controlled, maternal education, age, and socioeconomic factors were not analyzed. These should be considered in future studies, as they may influence neurological development [62,63]. In addition, post-discharge factors such as the type and adequacy of nutrition, access to early physiotherapy, physical activity levels, and the caregiving environment (e.g., home care vs. day care) were not systematically recorded or controlled for in this study. These variables can significantly influence long-term neurodevelopmental outcomes and may have contributed to the variability in BSID-III scores.

### 4.5. Implications for Clinical Practice and Future Research

While individualized TFO has demonstrated clinical advantages in improving growth, nutritional intake, and potentially neurodevelopmental outcomes in preterm infants, its implementation is associated with logistical and financial implications that must be acknowledged. TFO requires the integration of near-infrared breast milk analyzers, modular macronutrient components, trained personnel, and infrastructure for analysis and documentation. Previous studies have shown that analysis takes approximately 5–7 min per sample, and recipe calculation another 2–5 min, leading to an estimated workload of 10–15 min per infant per day [20]. The initial investment in equipment (e.g., milk analyzers costing EUR 40 k–60 k) is substantial, and ongoing costs include cleaning, maintenance, and staff time. However, this must be weighed against the potential to reduce complications associated with postnatal growth restriction, including prolonged NICU stay, neurodevelopmental impairments, and metabolic morbidities in later life [64]. Furthermore, the cost may be offset by the reduced need for catch-up growth strategies and improved long-term outcomes. Importantly, streamlined workflows and digital calculation tools (e.g., Excel-based recipes) have been shown to enhance operational feasibility in real-world settings. Thus, while TFO introduces initial and ongoing costs, its potential to standardize nutrition, reduce variability, and improve outcomes supports its consideration for integration into NICU practice, especially in centers with high volumes of preterm infants or existing milk management infrastructure.

We believe that TFO can be adapted and implemented in a variety of clinical settings, including lower-level NICUs and resource-limited environments. Simplified TFO models, which require only 2–3 milk measurements per week combined with digital recipe tools, have shown promising feasibility and efficacy. These streamlined approaches include batching expressed breast milk for 24 h feeding periods and basing fortification prescriptions on current milk analysis for the following one to two days. Modular supplements can be prepared using a practical, coin-like system, simplifying preparation and administration. Moreover, the maintenance demands of modern milk analyzers are relatively low, requiring only basic cleaning routines and periodic calibration with low and high standard values.

Importantly, the long-term benefits of individualized milk analysis and nutrient-targeted feeding, including improved growth, reduced morbidities, and potentially better neurodevelopment, could offset the initial investment and operational costs, making TFO a cost-effective intervention even in settings with limited resources.

According to current ESPGHAN guidelines for the nutritional management of preterm infants after discharge, nutritional support is required, if an infant leaves the hospital with undernutrition (defined as weight- and/or length-for-age (before term), or length-for-age and/or weight-for-length z-score (after term) below –2 standard deviations) or if poor postnatal growth is observed [65]. TFO prevents undernutrition, but there is a potential loss of nutritional benefit after discharge, particularly in infants who continue to receive breast milk with low protein content. This also underscores the need for long-term nutritional strategies in infants not showing undernutrition to maintain the positive effects of TFO. One promising approach involves extending TFO into the post-discharge period, typically covering the first four months corrected age until the introduction of complementary feeding. Although not yet implemented in clinical practice, the conceptual framework for home-based TFO is currently under development. It includes periodic analysis of expressed breast milk sent from home, digital tracking of growth trajectories, and remote adjustments to nutrient supplementation through established telemedicine platforms. A growth trajectory calculator tailored to preterm infants has already been developed and is in the process of being integrated into a mobile application designed for parental use. While the logistics of collecting and analyzing breast milk samples at a distance are still being explored, our research group is planning future multicenter studies to evaluate the feasibility, safety, and efficacy of this approach. It would allow for continued individualized nutrition aligned with ESPGHAN recommendations and would offer a promising path toward maintaining and enhancing the developmental benefits initiated during hospital-based TFO.

Future research should aim to validate the effects of TFO through larger trials conducted across multiple centers. Future studies should also explore practical strategies for incorporating TFO into everyday clinical workflows and examine its economic viability. Investigating the extension of TFO into the post-discharge period could help maintain the developmental advantages gained during NICU care. Further work is needed to refine fortification practices by enhancing the quality of nutrients used, including evaluating different protein sources, the addition of human milk oligosaccharides, and the optimization of micronutrient supplementation (e.g., calcium, vitamins, electrolytes). Understanding how TFO influences the gut microbiome and its relationship to systemic health and neurodevelopment represents another promising future area of inquiry.

## 5. Conclusions

TFO ensures that preterm infants receive balanced nutrition by measuring all three macronutrients in human milk and adding protein, fat, and carbohydrates as needed and recommended by ESPGHAN. Compared to the control group, the intervention group achieved significantly higher intakes of these macronutrients, a higher protein-to-energy ratio, greater body weight at 36 weeks, and faster growth velocity.

Subgroup analysis showed that preterm infants receiving low-protein human milk had significantly higher fat-free mass with TFO. While most BSID-III outcomes did not reach statistical significance, a trend toward improved neurological development was observed, including significantly better scores in the language subscale and fewer infants with motor scores ≤70. These findings, though encouraging, should be interpreted with caution due to the limited sample size and the exploratory nature of the subgroup analyses.

TFO’s effects may differ between short- and long-term outcomes, potentially due to post-NICU feeding with low-protein human milk. Identifying at-risk infants and optimizing post-discharge nutrition is crucial.

TFO is a promising approach to improving neurological development and reducing the risk of cognitive developmental delays. Multicenter studies are needed to increase the statistical power of the data, strengthen the evidence base, and evaluate the broader applicability and long-term benefits of this approach.

## Figures and Tables

**Table 1:** Baseline characteristics.

	Control Group			Intervention Group		
	All (n = 51)	BSID-III (n = 35)	No BSID-III (n = 16)	All (n = 52)	BSID-III (n = 34)	No BSID-III (n = 18)
Gestational age at birth (weeks)	27.3 ± 1.7	27.1 ± 1.8	27.7 ± 1.6	27.2 ± 1.2	27.2 ± 1.3	27.2 ± 1.0
Birth weight (g)	980 ± 270	970 ± 260	1010 ± 290	980 ± 210	950 ± 200	1030 ± 230
Length at birth (cm)	34.7 ± 3.7	34.9 ± 3.8	34.3 ± 3.8	35.2 ± 2.4	34.8 ± 2.5	36.1 ± 1.8
Head circumference at birth (cm)	24.6 ± 1.9	24.7 ± 2.2	24.5 ± 1.7	24.8 ± 1.7	24.6 ± 1.4	25.2 ± 2.2
Sex, number of males (percentage)	27 (53%)	19 (54%)	8 (50%)	28 (54%)	22 (65%)	6 (33%)
Apgar < 7 at 1 min	36 (71%)	24 (69%)	12 (75%)	35 (67%)	23 (68%)	12 (67%)
Apgar < 7 at 5 min	17 (33%)	10 (29%)	7 (44%)	14 (27%)	9 (26%)	5 (28%)
Maternal age (years)	29.7 ± 5.8	30.6 ± 5.2	27.8 ± 6.6	30.6 ± 6.3	29.8 ± 6.2	32.2 ± 6.3
PMA at start of study (weeks)	30.8 ± 1.4	30.6 ± 1.4	31.1 ± 1.5	30.5 ± 1.0	30.6 ± 1.1	30.3 ± 0.9
Day of life at start of study (days)	25 ± 7	25 ± 8	24 ± 7	23 ± 6	24 ± 7	22 ± 4
Duration of intervention (days)	28 ± 10	29 ± 11	26 ± 9	27 ± 9	29 ± 10	22 ± 5
Corrected age at BSID-III (months)		18.9 ± 1.9			18.5 ± 1.3	
Weight at BSID-III (kg)		11.1 ± 1.5			11.0 ± 1.3	
Length at BSID-III (cm)		80.8 ± 5.1			80.0 ± 3.2	
Head circumference at BSID-III (cm)		47.9 ± 1.8			47.6 ± 1.6	

“All” includes all preterm infants who completed the intervention; “BSID-III” includes all preterm infants who completed the intervention and were assessed using BSID-III. “No BSID-III” includes all preterm infants who completed the intervention but were not assessed using BSID-III due to loss to follow-up. Values are presented as the arithmetic mean with standard deviation or as absolute numbers (number of preterm infants for male sex and Apgar < 7 at 1 and 5 min). Intervention group versus control group were compared for “All”, “BSID-III”, and “No BSID-III”. No significant statistical differences were detected. n—number of preterm infants; Apgar—Apgar Score; PMA—postmenstrual age; BSID-III—Bayley Scales of Infant and Toddler Development III.

**Table 2:** Pregnancy risk factors and severe morbidities in the NICU.

	Control Group		Intervention Group	
	All (n = 51)	BSID-III (n = 35)	All (n = 52)	BSID-III (n = 34)
Maternal diabetes	3 (6%)	2 (6%)	6 (12%)	6 (18%)
Hypertension/preeclampsia	12 (24%)	7 (20%)	17 (33%)	15 (44%) *
Suspected chorioamnionitis	15 (29%)	10 (29%)	13 (25%)	9 (26%)
Antenatal corticosteroids	49 (96%)	34 (97%)	47 (90%)	29 (85%)
Deceased before discharge	2 (4%)	0 (0%)	0 (0%)	0 (0%)
NEC all cases	2 (4%)	0 (0%)	0 (0%)	0 (0%)
NEC Bell stage 3	1 (2%)	0 (0%)	0 (0%)	0 (0%)
Sepsis clinical	19 (37%)	15 (43%)	14 (27%)	14 (41%)
Sepsis culture positive	7 (14%)	6 (17%)	10 (19%)	7 (21%)
PDA	30 (59%)	22 (63%)	29 (56%)	21 (62%)
PDA treated	21 (41%)	14 (40%)	14 (27%)	9 (26%)
BPD mild	17 (33%)	10 (29%)	15 (29%)	10 (29%)
BPD moderate/severe	18 (35%)	15 (43%)	16 (31%)	14 (41%)
Feeding intolerance	14 (27%)	11 (31%)	8 (15%) **	5 (15%)

“All” includes all preterm infants who completed the intervention; “BSID-III” includes all preterm infants who completed the intervention and were assessed using BSID-III. Values are presented as absolute numbers (number of preterm infants with the respective characteristic), with percentages in parentheses indicating the proportion of infants with a given characteristic relative to the total number of infants in the respective subgroup. Comparisons were made between the control and intervention groups for “All” and “BSID-III” using chi^2^-test. Significance level: * *p* < 0.05; ** *p* < 0.01. BSID-III—Bayley Scales of Infant and Toddler Development III; n—number of preterm infants; NEC—necrotizing enterocolitis; PDA—patent ductus arteriosus; BPD—bronchopulmonary dysplasia.

**Table 3:** Macronutrient content of native human milk and fortified milk, duration of intervention, nutrient intake, and growth parameters.

	All			Low-Protein Group			High-Protein Group		
	Control Group (n = 35)	Intervention Group (n = 34)	Mean Difference [95% CI]	Control Group (n = 17)	Intervention Group (n = 20)	Mean Difference [95% CI]	Control Group (n = 18)	Intervention Group (n = 14)	Mean Difference [95% CI]
Native human milk									
Fat (g/dL)	3.6 ± 0.5	3.6 ± 0.6	0.0 [−0.3; 0.2]	3.4 ± 0.5	3.4 ± 0.6	0.1 [−0.4; 0.3]	3.8 ± 0.4	3.9 ± 0.6	0.1 [−0.4; 0.3]
Lactose (g/dL)	6.5 ± 0.4	6.7 ± 0.4	0.2 [−0.4; 0.0]	6.4 ± 0.4	6.7 ± 0.4 *	0.3 [−0.6; −0.1]	6.7 ± 0.5	6.7 ± 0.4	0.0 [−0.4; 0.3]
Protein (g/dL)	1.2 ± 0.2	1.1 ± 0.2	0.0 [−0.1; 0.1]	1.0 ± 0.1	1.0 ± 0.1	0.1 [−0.1; 0.0]	1.3 ± 0.2	1.3 ± 0.1	0.0 [0.0; 0.1]
Intervention parameters—Nutritional intake									
Duration of intervention (days)	29 ± 11	29 ± 10	0.3 [−5.3; 4.7]	27 ± 10	28 ± 10	0.9 [−7.7; 5.9]	31 ± 12	31 ± 9	0.4 [−8.1; 7.3]
Total fluid intake (mL/kg/d)	155 ± 5	152 ± 5 **	3.2 [0.9; 5.6]	155 ± 5	151 ± 6 *	4.3 [0.7; 7.9]	155 ± 4	153 ± 4	1.8 [−1.2; 4.8]
Fat (g/kg/d)	7.1 ± 0.7	7.6 ± 0.7 *	0.5 [−0.8; −0.1]	6.8 ± 0.8	7.3 ± 0.7 *	0.5 [−1.0; −0.0]	7.4 ± 0.5	8.0 ± 0.6 *	0.5 [−1.0; −0.1]
Carbohydrates (g/kg/d)	10.8 ± 0.8	13.6 ± 0.6 ***	2.8 [−3.1; −2.5]	10.6 ± 0.4	13.5 ± 0.6 ***	2.9 [−3.3; −2.6]	11.0 ± 1.0	13.7 ± 0.6 ***	2.7 [−3.3; −2.2]
Protein (g/kg/d)	3.6 ± 0.3	4.5 ± 0.2 ***	0.9 [−1.1; −0.8]	3.3 ± 0.2	4.5 ± 0.1 ***	1.2 [−1.3; −1.1]	3.9 ± 0.2	4.6 ± 0.2 ***	0.7 [−0.9; −0.6]
Energy (kcal/kg/d)	122 ± 8	141 ± 8 ***	19 [−23; −15]	117 ± 8	138 ± 7 ***	21 [−26; −16]	126 ± 6	145 ± 7 ***	19 [−23; −14]
Protein-to-energy ratio (g/100 kcal)	3.0 ± 0.2	3.2 ± 0.2 ***	0.3 [−0.4; −0.2]	2.8 ± 0.2	3.3 ± 0.2 ***	0.4 [−0.6; −0.3]	3.1 ± 0.2	3.2 ± 0.2	0.1 [−0.3; 0.1]
Intervention parameters—Growth outcomes									
Weight at 36 weeks PMA (g)	2283 ± 332	2514 ± 289 **	231 [−381; −81]	2134 ± 277	2525 ± 283 ***	391 [−578; −203]	2423 ± 324	2498 ± 308	75 [−306; 156]
Average 21-day growth velocity (g/kg/d)	19.2 ± 2.2	21.7 ± 2.3 ***	2.5 [−3.6; −1.4]	18.7 ± 2.3	21.4 ± 2.3 **	2.6 [−4.2; −1.1]	19.7 ± 2.2	22.2 ± 2.2 **	2.5 [−4.1; −0.9]
Nutritional efficiency (g/dL)	12.4 ± 1.6	14.3 ± 1.7 ***	1.9 [−2.7; −1.1]	12.1 ± 1.5	14.2 ± 1.9 ***	2.1 [−3.3; −1.0]	12.7 ± 1.6	14.5 ± 1.6 **	1.8 [−3.0; −0.6]

Values are presented as arithmetic mean of infants with standard deviation and mean difference with 95% confidence interval (CI); comparisons are made between the control and intervention groups. n—number of preterm infants; PMA—postmenstrual age. Significance level: * *p* < 0.05; ** *p* < 0.01; *** *p* < 0.001.

**Table 4:** Anthropometric data at discharge.

	All			Low-Protein Group			High-Protein Group		
	Control Group (n = 29)	Intervention Group (n = 22)	Mean Difference [95% CI]	Control Group (n = 14)	Intervention Group (n = 12)	Mean Difference [95% CI]	Control Group (n = 15)	Intervention Group (n = 10)	Mean Difference [95% CI]
Total body mass (g)	2318 ± 356	2506 ± 273 *	187 [−371; −4]	2142 ± 292	2529 ± 273 **	388 [−618, −158]	2482 ± 338	2477 ± 285	5 [−264, 274]
Length (cm)	43.1 ± 2.2	44.1 ± 1.7	1.0 [−2.2; 0.2]	42.3 ± 2.0	44.8 ± 1.9 **	2.5 [−4.1, −0.9]	43.9 ± 2.2	43.3 ± 1.3	0.5 [−1.0, 2.1]
Head circumference (cm)	31.4 ± 1.5	31.5 ± 1.4	0.0 [−0.8; 0.8]	30.6 ± 1.3	31.4 ± 1.4	0.9 [−2.0, 0.3]	32.3 ± 1.1	31.5 ± 1.5	0.8 [−0.3, 1.8]
Fat mass (%)	21 ± 6	23 ± 6	2.6 [−6.1; 0.9]	21 ± 6	24 ± 6	2.9 [−7.5, 1.7]	20 ± 7	22 ± 7	2.0 [−7.8, 3.7]
Fat mass (g)	483 ± 186	583 ± 181	101 [−205; 4]	451 ± 144	610 ± 191 *	159 [−295, −23]	512 ± 219	551 ± 173	39 [−210, 132]
Fat mass index (kg/m^2^)	2.6 ± 0.9	3.0 ± 1.0	0.4 [−1.0; 0.1]	2.5 ± 0.8	3.1 ± 1.1	0.6 [−1.4, 0.1]	2.6 ± 1.1	2.9 ± 0.9	0.2 [−1.1, 0.6]
Fat-free mass (%)	79 ± 6	77 ± 6	2.6 [−0.9; 6.1]	79 ± 6	76 ± 6	2.9 [−1.7, 7.5]	80 ± 7	78 ± 7	2.0 [−3.7, 7.8]
Fat-free mass (g)	1835 ± 274	1922 ± 217	87 [−230; 55]	1691 ± 240	1920 ± 183 *	229 [−404, −54]	1970 ± 238	1925 ± 263	44 [−165, 254]
Fat-free mass index (kg/m^2^)	9.7 ± 0.9	9.9 ± 1.0	0.1 [−0.7; 0.4]	9.3 ± 0.8	9.7 ± 0.9	0.5 [−1.1, 0.2]	10.2 ± 0.8	10.0 ± 1.2	0.2 [−0.6, 1.0]
Body mass index (kg/m^2^)	12.3 ± 1.2	12.9 ± 1.4	0.6 [−1.3; 0.2]	11.7 ± 1.2	12.8 ± 1.7	1.1 [−2.3, 0.1]	12.8 ± 1.1	12.9 ± 1.2	0.1 [−1.0, 0.9]

Values are presented as arithmetic mean with standard deviation and mean difference with 95% confidence interval (CI); comparisons are made between the control and intervention groups; n—number of preterm infants. Significance level: * *p* < 0.05; ** *p* < 0.01.

**Table 5:** BSID-III scores.

	All				Low-Protein Group				High-Protein Group				Low-Protein Control vs. High-Protein Intervention	
Bayley Score	Control Group (n = 29)	Inter-vention Group (n = 22)	Mean Difference [95% CI]	*p*	Control Group (n = 14)	Inter- vention Group (n = 12)	Mean Difference [95% CI]	*p*	Control Group (n = 15)	Inter-vention Group (n = 10)	Mean Difference [95% CI]	*p*	Mean Difference [95% CI]	*p*
Cognitive	95.7 ± 13.0	99.3 ± 11.6	3.6 [−9.5, 2.4]	0.235	93.5 ± 15.9	97.0 ± 12.3	3.5 [−12.9, 5.9]	0.459	97.8 ± 9.6	102.5 ± 10.0	4.7 [−11.8, 2.4]	0.184	9.0 [−19.0, 0.6]	0.066
Language	87.4 ± 16.7	90.1 ± 14.8	2.7 [−10.7, 5.2]	0.497	89.1 ± 20.9	87.4 ± 17.5	1.7 [−11.5, 14.9]	0.796	85.8 ± 11.6 *	94.5 ± 7.9 *	8.8 [−16.8, −0.7]	0.034	5.4 [−17.4, 6.5]	0.353
—receptive	92.3 ± 15.3	93.9 ± 16.3	1.6 [−9.5, 6.3]	0.694	92.3 ± 19.1	91.1 ± 18.8	1.3 [−12.0, 14.6]	0.846	92.4 ± 11.7	98.1 ± 11.1	5.7 [−14.4, 2.9]	0.186	5.7 [−18.1, 6.6]	0.349
—expressive	84.8 ± 14.6	88.3 ± 10.7	3.4 [−9.9, 3.0]	0.289	86.3 ± 18.4	87.0 ± 12.7	0.7 [−11.3, 10.0]	0.900	83.4 ± 10.3 *	90.4 ± 6.2 *	7.0 [−13.9, −0.1]	0.048	4.1 [−14.7, 6.6]	0.431
Motor	93.0 ± 12.8	94.8 ± 9.2	1.8 [−7.2, 3.6]	0.501	91.5 ± 16.5	92.0 ± 8.9	0.5 [−10.0, 9.0]	0.914	94.3 ± 8.5	98.8 ± 8.2	4.5 [−10.6, 1.6]	0.142	7.3 [−17.0, 2.4]	0.133
—fine	97.3 ± 11.0	98.4 ± 10.6	1.1 [−6.4, 4.2]	0.676	96.3 ± 11.7	95.8 ± 11.2	0.6 [−7.3, 8.5]	0.882	98.1 ± 10.7	102.1 ± 8.7	4.1 [−11.3, 3.1]	0.256	5.8 [−13.7, 2.1]	0.144
—gross	89.2 ± 12.9	92.2 ± 9.4	3.0 [−8.5, 2.5]	0.286	87.3 ± 16.2	90.0 ± 10.3	2.7 [−11.8, 6.5]	0.556	90.8 ± 9.6	95.4 ± 7.2	4.5 [10.8, 1.8]	0.152	8.0 [−17.7, 1.6]	0.098

Values are presented as arithmetic mean with standard deviation and mean difference with 95% confidence interval (CI). n—number of preterm infants. Significance level: * *p* < 0.05.

**Table 6:** Number of preterm infants with BSID-III scores ≤85 and ≤70.

BSID-III	Control Group (n = 35)	Intervention Group (n = 34)	*p*
Cognitive scale ≤ 85	8	3	0.052
Cognitive scale ≤ 70	3	1	0.155
Language scale ≤ 85	14	11	0.250
Language scale ≤ 70	4	3	0.360
Motor scale ≤ 85	9	6	0.189
Motor scale ≤ 70	3	0 *	0.035

Values are presented as absolute numbers (number of preterm infants with the corresponding score); BSID-III—Bayley Scales of Infant and Toddler Development III; n—number of preterm infants. Significance level: * *p* < 0.05.

## Data Availability

Raw data are unavailable due to ethical restrictions.

## Abbreviations

The following abbreviations are used in this manuscript:

BM	Breast milk
BPD	Bronchopulmonary dysplasia
BSID-III	Bayley Scales of Infant and Toddler Development III
CI	Confidence interval
ESPGHAN	European Society for Paediatric Gastroenterology, Hepatology and Nutrition
GCLP	Good Clinical and Laboratory Practice
IVH	Intraventricular hemorrhage
NEC	Necrotizing enterocolitis
NICU	Neonatal intensive care unit
PDA	Patent ductus arteriosus
PMA	Postmenstrual age
RCT	Randomized controlled trial
SF	Standard fortification
TFO	Target fortification
